# Post-translational modification: a strategic response to high temperature in plants

**DOI:** 10.1007/s42994-021-00067-w

**Published:** 2022-02-15

**Authors:** Danlu Han, Zhibo Yu, Jianbin Lai, Chengwei Yang

**Affiliations:** Guangdong Provincial Key Laboratory of Biotechnology for Plant Development, School of Life Science, South China Normal University, Guangzhou, 510631 China

**Keywords:** Thermotolerance, Thermomorphogenesis, Post-translational modification, Plant

## Abstract

With the increasing global warming, high-temperature stress is affecting plant growth and development with greater frequency. Therefore, an increasing number of studies examining the mechanism of temperature response contribute to a more optimal understanding of plant growth under environmental pressure. Post-translational modification (PTM) provides the rapid reconnection of transcriptional programs including transcription factors and signaling proteins. It is vital that plants quickly respond to changes in the environment in order to survive under stressful situations. Herein, we discuss several types of PTMs that occur in response to warm-temperature and high-temperature stress, including ubiquitination, SUMOylation, phosphorylation, histone methylation, and acetylation. This review provides a valuable resolution to this issue to enable increased crop productivity at high temperatures.

## Introduction

Climate change has resulted in a significant increase in the average global temperature and has rapidly changed the farming environment over the past few years (Teixeira et al. 2013; Yang et al. 2017). Temperature not only affects the metabolic balance of crops, but high temperature also accelerates the risk of drought stress on the metabolism of crops and limits photosynthetic efficiency. Short-term exposure to extreme temperatures can also greatly decrease the yield of crops, especially in critical development stages, where heat stress damage is particularly severe (Sun et al. 2019; Tigchelaar et al. 2018; Zhao et al. 2017). During their long-term evolution, plants have developed regulatory networks and adaptive mechanisms at the physiological, biochemical, and cellular-molecular levels in response to the surrounding environment (Li et al. 2018). In the post-translational modifications (PTMs) of proteins, a small molecule is added to modify the location, stability, or function of target proteins, which is vital for survival in acute stress situations because it enables a swift response to a changing environment (Millar et al. 2019).

Plants have evolved a set of defense strategies to adequately respond to the complexity and diversity of environmental temperature changes. In response to high-temperature stress, plants perceive and transmit signals through receptors, including a plasma membrane channel, a histone sensor in the nucleus and cytosol, and two unfolded protein sensors in the endoplasmic reticulum (ER). Subsequently, transcription factors (TFs) in the regulatory network initiate the expression of downstream genes by binding to cis-acting elements of downstream target genes to enhance plant heat tolerance (Saini et al. 2021). Even though there has been extensive research over the past several decades, transcriptional regulation continues to be intensely scrutinized. The regulatory mechanism depends on the intensity of the heat and the duration and speed of the temperature increases. When the temperature increases 5–6 °C above the optimum ambient temperature, this mainly affects plant growth and development of hypocotyls and petioles, as well as flowering. Respiration and photosynthesis change when the temperature increases at this stage. Thus, in the plant's response to early heat stress, the network of bHLH transcription factor phytochrome-interacting factor 4 (PIF4) acts as a central mechanism of thermomorphogenesis (Choi and Oh 2016; Li et al. 2018; Quint et al. 2016). As temperatures continue to increase and harm plant life, they produce heat shock (HS). At the HS stage, emerging evidence indicates that heat shock factor (HSF)-dependent transcriptional networks regulate thermoresponsive gene expression. In addition, calcium (Ca^2+^) signaling, reactive oxygen species (ROS) signaling, nitric oxide (NO) signaling, and the unfolded protein response (UPR) are also involved in the plant response to HS (Ding et al. 2020; Li et al. 2018).

Perception and transduction of extracellular signals are essential for the survival of organisms. For the environment that is undergoing long-term change, cells can reprogram gene expression to respond to external stimuli, but for short-term stress, cells must adapt to stimuli by altering the activity and function of existing proteins. To perform this rapid regulatory mechanism, cells evolved by creating diversified small label tools, including phosphoric acid, acetyl groups, lipids, and other small peptides. When combined with their target proteins, these labels can quickly coordinate the behavioral change of their target proteins to respond to the complex environmental changes. The process of labeling proteins is called PTM (Beltrao et al. 2013). Most PTMs are not present on the given target proteins, which denotes that they enable rapid improvement of the modified proteins through the action of modifying enzymes. Importantly, this process is independent of energetically expensive protein synthesis and degradation and work-saving (Olsen and Mann 2013).

The PTM further promotes complexity and variety from the genome to proteome by regulating the activity, location of proteins, and their interaction with other proteins or nucleic acids. To date, more than 400 types of post-translational modifications have been identified, and they are involved in regulation throughout cell life activities (Bateman et al. 2017). It is estimated that 5% of the proteome is composed of their enzymes, including kinases, phosphatases, transferases, and ligases. These enzymes are mainly used to add and remove small peptides in the formation of amino acids, or cleave peptide bonds to remove specific sequences for forming a specific modifying molecule. In addition to these functions, they also modify themselves by autocatalytic domains for regulating their own activity (Walsh 2006). There has been a rapid progress over the past decade in the research into plant PTMs, with analytical approaches that have been improved, and further elaboration of their functions and regulatory mechanisms in plants. Herein, we focus on five types of PTMs in response to high-temperature in plants.

## Ubiquitination regulates protein stability in response to high temperature

The ubiquitin (Ub) protein was first discovered in 1975, and it covalently binds to the lysine residues of its target protein, and then targets the substrate for proteasomal degradation (Hershko and Ciechanover 1998). Ub was identified as being responsible for modulating protein turnover and homeostasis by the addition of one or more ubiquitins in different configurations. This provides system flexibility and diversity in the response to different environmental conditions by plants (Sadanandom et al. 2012). Although there are few studies on ubiquitination in plants under high-temperature stress, nevertheless, it has been shown that Ub plays an important role in modulating plant response to temperature. Ub is preceded by an enzymatic conjugation cascade, including three types of enzymes: ubiquitin-activating enzyme E1, ubiquitin-conjugating enzyme E2, and ubiquitin ligase E3. E3 ligase facilitates the transfer of ubiquitin to the substrate protein due to its capacity to recognize the target proteins (Callis 2014). According to the conjugated ubiquitin's quantity and position, there are three types of ubiquitination: monoubiquitination, multi-monoubiquitination, and polyubiquitination (Deshaies and Joazeiro 2009). The first two types mainly act as markers for protein kinase activity (Salghetti et al. 2001; Feng and Shen 2014; Ma et al. 2020). A chain of more than four ubiquitins is conjugated to a substrate protein, and the targets may direct the substrate to the 26S proteasome for proteolysis (Thrower et al. 2000).

Accumulating evidence indicates that the E3 ligase genes are induced by HS, and gene overexpression can increase the thermotolerance of plants. In *Arabidopsis*, *AtPUB48* expression was induced by heat stress, and is involved in basal and acquired thermotolerance. Overexpression of AtPUB48 resulted in increased heat tolerance during seed germination and seedling growth (Peng et al. 2019). Another *Arabidopsis* conserved chaperone-dependent ubiquitin E3 ligase, carboxyl terminus of HSC70-interacting proteins (CHIP), also exhibits a similar function upon heat stress (Yan et al. 2003). Importantly, the function of ubiquitin E3 ligase is conserved in plants. The expression of *Solanum lycopersicum* CHIP (SlCHIP) was induced under high temperature in tomatoes, and silencing of SlCHIP in tomatoes decreased heat tolerance (Zhang et al. [Bibr CR112][Bibr CR113]). These results indicate that the Ub ligase CHIP plays a critical role in plant heat stress responses. In rice, researchers found RING E3 ligase localized in the Golgi apparatus, and heat- and cold-induced 1 (OsHCI1) accumulated in the nucleus and interacted with nuclear substrate proteins under high temperature. Overexpressed OsHCI1 in *Arabidopsis* plants enhanced thermotolerance (Lim et al. 2013). Similarly, overexpression of the *Sorghum bicolor* orthologue SbHCI1 in *Arabidopsis* also improved basal heat stress tolerance. Interestingly, under heat stress conditions, it was observed that SbHCI1 migrated to the Golgi bodies from the cytoplasm (Lim et al. 2020). Therefore, it suggests that *HCI1* may have evolved independently of the two speciation to result in differences. In addition to HCI1, another RING finger ubiquitin E3 ligase, heat tolerance at seedling stage (OsHTAS), also plays a positive role in heat tolerance at the seedling stage. Previous studies found that OsHTAS could interact with components of the ubiquitin/26S proteasome system and an isoform of rice ascorbate peroxidases. Further studies proved that OsHTAS enhances heat tolerance through modulation of hydrogen peroxide-induced stomatal closure (Liu et al. 2016). On the contrary, drought, heat, and salt-induced *Oryza sativa* ring finger E3 ligase, ring finger protein 1 (OsDHSRP1), acts as a negative regulator. Although the OsDHSRP1 gene transcripts were highly expressed under heat stress conditions, overexpressing OsDHSRP1 in *Arabidopsis* resulted in hypersensitivity to heat stress (Kim et al. 2020). The above research results suggested that the function of E3 ligases is diverse and complex.

In addition to the ubiquitin-proteasome system, ubiquitin also modifies some key TFs that enable plant adaptation to heat stress. It was found that dehydration-responsive element binding protein 2A (DREB2A) is a key transcription factor that regulates the expression of many drought- and heat-stress-inducible genes in *Arabidopsis*. Morimoto et al. identified BTB/POZ and math domain proteins (BPMs), which are substrate adaptors for a Cullin3-based E3 ubiquitin ligase as a DREB2A-interacting protein. The researchers also found that BPMs were involved in DREB2A degradation under heat stress conditions. The results of genetic analysis and biochemical assays indicated that the depletion of *BPM* expression contributed to thermotolerance via DREB2A stabilization. They also suggested that E3 ubiquitin ligase BPMs modulate the heat stress response and prevent any  adverse effects of excess DREB2A on plant growth (Morimoto et al. 2017).

**Fig. 1:**
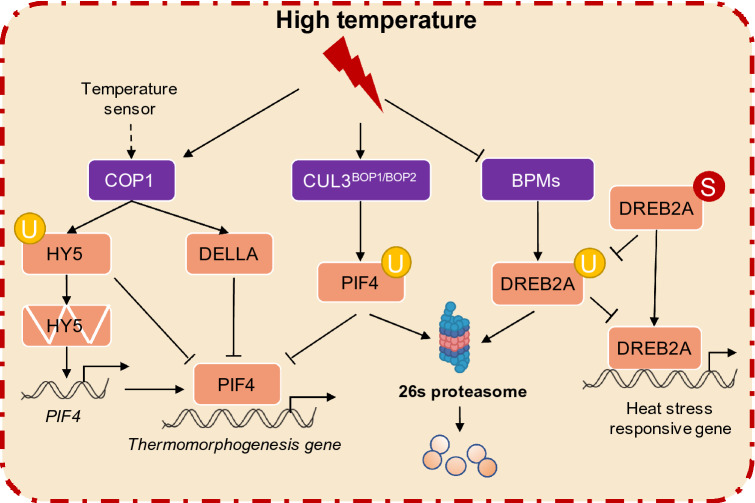
The regulation of ubiquitination in high-temperature stress responses in plants. Under warm temperatures, COP1 accumulates in the nucleus and degrades HY5 via ubiquitination to further enhance PIF4 activity. COP1 then interacts with DELLA proteins and increases the activity of DELLA to promote growth under the same conditions. DELLA can attenuate PIF4-mediated thermomorphogenesis activity under long-day conditions. PIF4 was ubiquitinated by the CUL3BOP1/BOP2 E3 ubiquitin ligase complex for further degradation. Under normal conditions, DREB2A is ubiquitinated by BPMs and degraded. Upon heat shock, SUMOylation maintains the protein stability of DREB2A, which regulates the downstream heat stress response genes

Under warm temperatures, ubiquitination controls another regulation mechanism. The E3 ubiquitin ligase constitutive photomorphogenic 1 (COP1), the master regulator of photomorphogenesis, is regulated primarily at the protein level in response to external stimuli (Lau and Deng 2012). Because warm temperatures could induce hypocotyl elongation, the researchers found that COP1 modulates hypocotyl thermomorphogenesis, based on tracking information flow. They further explained that COP1 is located in the nucleus, and promotes the degradation of the thermomorphogenic repressor HY5, and stabilizes PIF4 under warm temperatures (Legris et al. 2017; Martínez et al. 2018; Park et al. 2017). Additionally, COP1 physically interacts with transcriptional regulators DELLA proteins, which serve as signaling hubs between the environment and the transcriptional networks in the control of plant growth. At 28°C, COP1 degrades DELLA proteins (Blanco-Touriñán et al. 2020). As we previously mentioned, PIF4 plays a key role in the regulated network of warm temperatures. In a recent study, the CUL3-based E3 ligase (CUL3^BOP1/BOP2^) and high expression of osmotically responsive genes 1 (HOS1) were shown to interact with PIF4 (Kim et al. 2017; Zhang et al. 2017a). The E3 ligase complex CUL3^BOP1/BOP2^ mediates PIF4’s protein ubiquitination and degradation in response to temperature (Fig. 1). For another E3 ligase HOS1, it was determined that it suppresses the transcriptional activity of PIF4 (Kim et al. 2017). Recently, Zhang et al. found another E3 ligase, XB3 ortholog in *Arabidopsis thaliana * (XBAT31), that acts as a positive thermomorphogenesis regulator and mediates the protein stability of ELF3 to affect hypocotyl growth under warm temperatures (Zhang et al. 2021a). These studies demonstrate that PTMs play an important role in plant temperature responses.

Except for heat stress response (HSR), UPR is also an essential mechanism in response to heat stress in plants (Malini et al. 2020). Accumulation of misfolded proteins induced by high temperature activates the UPR to alleviate ER stress (Strasser 2018). When the UPR pathways are blocked, the misfolded proteins are ubiquitinated for degradation through ER associated degradation (ERAD) pathway. Ubiquitination is a vital step in the ERAD pathway (Deng et al. 2013). In plants, the core components of the ERAD pathway were identified, such as membrane-anchored ubiquitin E3 ligase brassinosteroid insensitive 1 suppressor 7 (EBS7), HMG-CoA reductase degradation1 a/b (Hrd1a/b), protein associated with Hrda1-1/2 (PAWH1/2) and ERAD-mediating ring finger (EMR) (Liu et al. 2015; Chen et al. 2016b; Park et al. 2018; Lin et al. 2019). The molecular mechanism of the ERAD pathway still remains poorly understood in plants, although the components are conservated in eukaryotic cells. At present, the heat-responsive studies of canonical URP regulators mainly focus on the bZIP family and NAC family in plants. During HS, the transcriptional factor bZIP28 directly targets heat stress response genes, and it was observed that the *bzip28bzip60* double mutant was sensitive to HS (Zhang et al. 2017b). Similarly, in rice, OsbZIP74 is also pivotal for heat stress tolerance. Under heat stress, Osbzip74 increases gene expression of the NAC transcription factor OsNTL3, which regulates the expression of downstream genes in response to heat stress and the ER stress. Further OsNTL3 transmits heat stress signals/effects from the plasma membrane to the nucleus (Liu et al. 2020).

In conclusion, ubiquitination plays important roles in mediating the accurate changes required for growth and development as well as adaption to environmental stresses. Mono-ubiquitination or multi-mono-ubiquitination, activates a variety of their functions, such as activity, transcription regulation, trafficking, subcellular localization and signal transduction (Hicke and Dunn 2003; Wu et al. 2003). Under high-temperature conditions, the main function is the poly-ubiquitination of target proteins, which degraded unfolded proteins via the 26S proteasome. Of these, the greatest amount of study has been devoted to E3 ligases, but there are other related proteins of the ubiquitin system, such as the  α2 subunit of the 26S proteasome TT1 (Thermo-tolerance 1) gene that markedly enhances the thermotolerance in rice (Li et al. 2015). Despite this, current research on molecular functions, and mechanisms of action for the ubiquitination response to heat stress remains at a rudimentary stage. Therefore, it is necessary to identify the Ubiquitination substrate proteins of important crops, especially using global genome-level analyses.

## SUMOylation rapidly controls transcription under high temperature

Small ubiquitin-like modifiers (SUMOs) are ubiquitin-like polypeptides  that are also involved in the heat-stress response. The enzymatic mechanism of SUMOylation is similar to that of ubiquitination, whereby C-terminal Gly is covalently attached to accessible Lys in the target by a protein conjugation cascade. SUMO precursors are proteolytically processed to expose their C-terminal double glycine (Gly-Gly) motifs by SUMO-specific proteases, and activated SUMO is driven by a SUMO activating enzyme 1 (SAE1, E1) in an ATP-dependent reaction. Then, the activated SUMO is transferred to the SUMO-conjugating enzyme 1 (SCE1, E2) and diverted to substrate proteins with assistance from a SUMO-protein ligase (E3) by transesterification (Saracco et al. 2007).

Under acute high temperature (37°C/42°C), the levels of SUMO conjugates are markedly increased in response to stress. This is described as a conserved “SUMO stress response” (SSR) in eukaryotes (Lewicki et al. 2015), and it supports  the eukaryotic response to acute stress. In *Arabidopsis*, fewer SUMO1/2 conjugates were detected with overexpression of HSP70 during heat shock (Kurepa et al. 2003), suggesting that the accumulation of SSR is relevant to HSR. Temperatures around 27-28°C are referred to as warm temperatures, which do not lead to up-regulation of known heat stress marker genes and permanent protein damage in plants (Kumar and Wigge 2010). In relation to heat stress, plants respond to slightly elevated temperatures for a longer period of time mainly via thermal morphogenesis, like petiole elongation, leaf hypophysis, and early flowering (Qiu et al. 2019; Quint et al. 2016). A SUMO1/2 knockdown mutant exhibited thermosensitivity at 28°C. Van den Burg et al. found that SUMO-dependent thermo-resilience can be potentially controlled in a different manner. SUMO1/2 activates temperature acclimation via the master regulator of heat stress: the HSFA1 family (Hammoudi et al. 2021). This finding explained that SUMO1/2 have key roles in enabling plants to withstand prolonged warm periods. Similar to ubiquitination, overexpression of SUMO E3 ligase (such as AtSIZ1, OsSIZ1, SlSIZ1 and GmSIZ1) enhanced plants tolerance to heat stress (Cai et al. 2017; Mishra et al. 2018; Yoo et al. 2006; Zhang et al. 2018). AtSIZ1 is also a positive regulator of thermorphogenesis upstream of the PIF4 regulation hub by cross-talking with ubiquitin E3 ligase COP1 (Hammoudi et al. 2018).

**Fig. 2:**
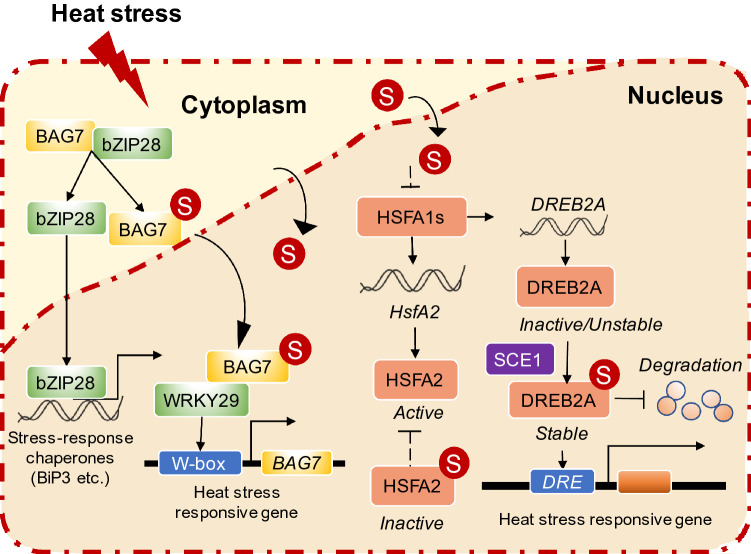
The molecular mechanism of the SUMO stress response under heat shock. Under HS, the SUMO stress response (SSR) is triggered by a large amount of SUMO1/2 entering from the cytoplasm into the nucleus in plant cells. To date, researchers determined that the regulatory mechanisms consist of BAG7 interacting with BiP2 and bZIP28 in the ER under normal conditions. When HSR and UPR are activated, BAG7 and bZIP28 dissociate with BiP2, and BAG7 is SUMOylated. SUMOlyted BAG7 translocates to the nucleus to interact with WRKY29 and induce the gene expression of stress-response chaperons. bZIP28 translocates from the cytoplasm to the nucleus to induce expression of *BiP3* by UPR. The HSFA2 is SUMOylated, leading to further decreases in its transcriptional activation. The SUMO conjugation of DREB2A prevents its interaction with the ubiquitin ligase BPMs, enhancing the stabilization of the TF, and ultimately leading to the promotion of heat-induced gene expression

Proteome-wide studies in *Arabidopsis* and maize revealed that HS induces SSR, particularly on the target proteins in the nucleus, which includes the response to stress by TFs (the HSF, NAC and WRKY families), chromatin remodeling complexes (SWI/SNF complexes), and RNA-related proteins (RNA-splicing factor, RNA helicase) (Miller et al. 2013; Rytz et al. 2018). They suggested that SUMOylation regulates the heat stress response at different levels, including transcription, post-transcriptional processing, and translation. Current evidence indicates that there are several important TFs involved in HS responses. When heat stress occurs, it induces increased accumulation of misfolded proteins in cells. BAG7 (B-cell lymphoma 2 (Bcl2)-associated athanogene (BAG) proteins) interacts with bZIP28 anchored to the ER membrane. The SUMO attachment on BAG7 contributes to its translocation to the nucleus for the enhancement of gene expression in the UPR during HS by interaction with WRKY29 (Li et al. 2017). SUMOylation of a heat shock transcription factor, AtHSFA2, is essential for HSR and acquired thermotolerance (Cohen-Peer et al. 2010).  DREB2A interacts with SCE1, a SUMOylation component, and consequently increases DREB2A protein stability under high temperature (Wang et al. 2020). Thus, in contrast to ubiquitination, SUMOylation mainly maintains the stability of substrate proteins for heat-stress response (Fig. 2).

In addition, as high-throughput sequencing technology has evolved the concept of group SUMOylation has emerged, i.e. coordinated SUMO modification of multiple proteins of a functional protein complex, which is thought to enhance protein-protein interactions within the complex by SUMO-SIM pairing (Niskanen and Palvimo 2017). SUMOylation acts in a novel role, mediating the transcription switch mechanism between development and HS in plant cells by targeting different proteins (Han et al. 2021). The interconnected dual role for SIZ1 and SUMO1/2 conjugation in the switch between plant immunity and high temperature-induced growth also supports this view (Hammoudi et al. 2018, 2021). In general, SUMOylation is vital for survival in stressful situations because it enables a swift reaction to a changing environment.

## Phosphorylation cascades regulate signaling transduction under high temperature

Protein phosphorylation is a major post-translational modification that influences protein activity, interaction, localization, and stability. Phosphorylation is a highly dynamic and reversible process mediated through kinases and phosphatases, which often serve as 'on-and-off’ switches in the regulation of many cellular activities (Luan 2003). The *Arabidopsis* genome encodes more than 1000 protein kinases, including mitogen-activated protein kinases (MAPKs), sucrose non-fermenting-related kinases (SnRKs), calcium-dependent protein kinases (CPKs) and receptor-like kinases (RLKs). Compared to protein kinases, the *Arabidopsis* genome encodes only approximately 150 protein phosphatases, which include protein phosphatase 1 (PP1) and PP2A-type phosphatases, the protein tyrosine phosphatase family, and the metal-dependent protein phosphatase family (Kerk et al. 2008). Accumulating evidence has shown that phosphorylation acts as a central participant in the regulation of gene expression to allow rapid adaptation of plants to environmental stress (Zhu 2016). The functional roles of phosphorylation in plant thermal response are summarized below.

Cyclin-dependent kinase CDC2s interact with *Arabidopsis* HSF1, a master regulator for the expression of HSPs and thermotolerance, and they phosphorylate AtHSF1 in vitro. This inhibits binding to the heat-shock elements of target DNA, suggesting a possible regulatory relationship between heat stress response and cell-cycle control in plants. However, the mechanism used by Cdc2a to prevent the DNA-binding activity of AtHSF1 remains unclear (Reindl et al. 1997). Further experiments showed that *Arabidopsis* CaM-binding protein kinase 3 (AtCBK3) participates in fundamental thermotolerance and that it interacts with and phosphorylates AtHSFA1, which regulates the binding to HSEs and the expression of HSP genes during heat stress (Liu et al. 2008). Furthermore, AtCBK3 activity can be stimulated by AtCaM. Importantly, AtCaM3 regulates the DNA-binding activity of HSFs to adjust plant thermotolerance. Therefore, AtCaM3 involved in plant thermotolerance is related to the AtCBK3-mediated phosphorylation status of HSFA1 (Zhang et al. 2009). In addition, *Arabidopsis* PP7, a Ser/Thr phosphatase, interacts with AtCaM3 and AtHSF1, and is also involved in heat stress through mediating the expression of AtHSP70 and AtHSP101 (Liu et al. 2007). Previous research showed that HSFA1s directly modulate the expression of transcription factors that are important in the heat stress response, including the induction of DREB2A (Praat et al. 2021). DREB2A is a key transcriptional activator that induces many heat- and drought-responsive genes involved in the stress process. DREB2A is unstable under non-stress conditions, but stabilizes in response to stress. Recently, Mizoi et al. found that DREB2A is a substrate of CK1, and the level of phosphorylation is reduced in response to stress. The accumulation of DREB2A led to the downstream stress-responsive genes expression that accounts for the enhanced thermotolerance (Mizoi et al. 2019).

In addition to HSFA1, other *Arabidopsis* HSFA members are also phosphorylated by Mitogen-Activated Protein Kinases (MAPKs/MAP kinases), which modulate the plant response to heat stress. Alfalfa cells have two temperature-responsive MAPKs, SAMK (Stress-Activated MAP Kinase) and HAMK (Heat Shock-Activated MAP Kinase), which are activated by a relative temperature shift, suggesting that MAP kinases have a critical role in regulating thermotolerance in plants (Sangwan and Dhindsa 2002). *Arabidopsis* mitogen-activated protein kinase MPK6 is induced by heat stress and phosphorylates several heat stress transcription factors, but strongly phosphorylates HSFA2, and thereby contributes to regulating heat stress-induced nuclear accumulation of HSFA2. In addition, HSFA2 protein stability is regulated in a phosphorylation-dependent manner, but it is independent of MPK6, indicating that the activity of HSFA2 is subject to multiple regulatory mechanisms (Evrard et al. 2013). MPK6 and MPK3 also interact with and phosphorylate the major Ser-309 residues of HSFA4, which if mutated, strongly diminish the activity of heat shock protein 17.6A (HSP17.6A). Interestingly, HSFA4A, as a substrate of MPK3/MPK6, modulates plant response to salt and oxidative stress (Pérez-Salamó et al. 2014). Further research demonstrated that MAP kinases, such as MPK3, MPK4, and MPK6, phosphorylate HSFA4A in the Ser-309 residue as the dominant phosphorylation site, indicating that heat-activated factor HSFA4A is regulated by a complex mechanism. In addition, HSFA4A becomes activated by high salinity or high temperature, and a combination of these conditions regulates the abiotic stress response by increasing the accessibility of the HSFA4A binding site at the target gene promoters, *ZAT12*, *HSP17.6A*, and *WRKY30* (Andrási et al. 2019). After salt stress treatment, HSFA4A is transported into the nuclei,  which is in accordance parallel with a previous report and indicates that HSFs accumulate in the nuclei upon heat and other stresses. Tomato MAP kinase also can be activated upon heat stress. Heat-activated MAP kinase phosphorylates and promotes HSFA3 expression, which is calcium-dependent (Link et al. 2002). MAP kinases in mammals and yeasts have been found to be involved in heat-stress signaling through phosphorylating HSFs, which indicates that MAP kinases  that modulate the heat stress response are conserved in eukaryotes (Link et al. 2002). The biological or molecular function of such MAP kinase-mediated phosphorylation, however, requires further confirmation.

**Fig. 3:**
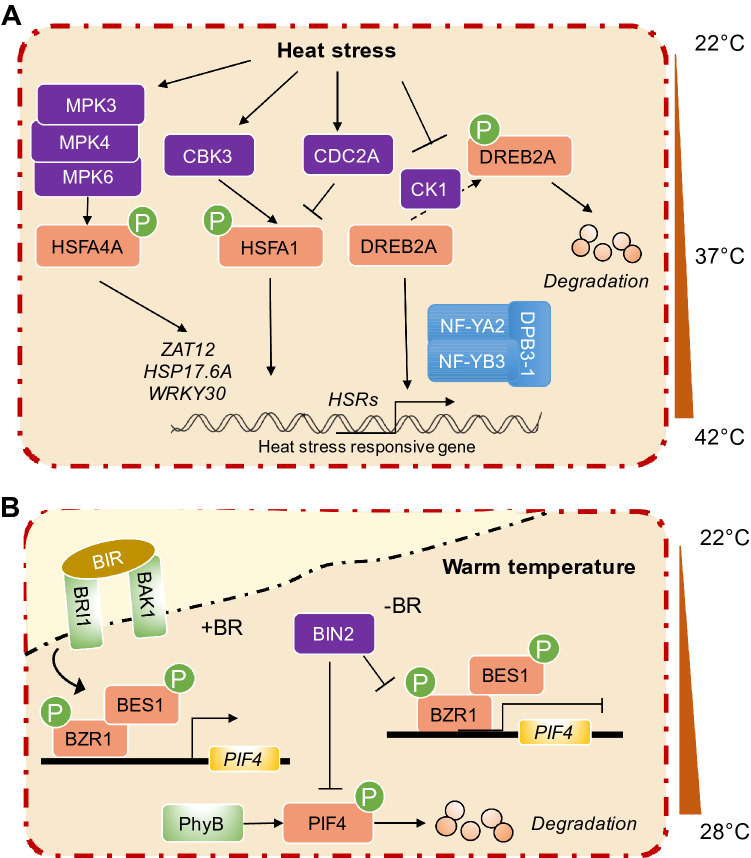
Phosphorylation regulates the response by kinases to high temperatures. **A** Under HS, heat stress-response transcriptional factors were found as phosphorylated substrates. HSFA4A interacts with kinases MPK3/4/6 in plant cells, which phosphorylate HSFA4A to activate the downstream heat stress-response genes: ZAT12, HSP17.6A, and WRKY30. HSF1 is phosphorylated, which CBK3 that is involved in the stress response. Nevertheless, kinase CDC2A mediates the phosphorylation of HSF1, which inhibits the binding of HSF1 to chromatin. Under normal conditions, casein kinase 1 (CK1) mediates the phosphorylation of DREB2A, which promotes the degradation of DREB2A. Under HS, DREB2A proteins are dephosphorylated and SUMOylated to maintain protein stability. Then, DPB3.1 binds to the transcription factor DREB2A, forms a heterotrimer with NF-YA2 and NF-YB3 under high-temperature stress conditions, and binds to DREB2A to form a transcriptional complex bound to the promoter region of stress-related target genes, thus enhancing the transcriptional activity of DREB2A to activate downstream HSR genes. **B** The BZR1/BES1 is phosphorylated by heterodimers BZR1/BES1 to activate PIF4 transcription in the presence of BR under warm temperatures. For kinase BIN2, warm temperature could increase phosphorylation of PIF4, which interacts with phyB for degradation. And BIN2 also phosphorylates BZR1 and BES1 that prohibit transcription of *PIF4*

Phosphorylation events have been implicated in the regulation of plant thermomorphogenesis. The bHLH transcription factor PIF4 acts as a central integrator of plant thermomorphogenesis. The PIF4 factor was phosphorylated by the BR signaling kinase brassinosteroid insensitive 2 (BIN2), which marks PIF4 for proteasome degradation to contribute to diurnal hypocotyl growth (Bernardo-García et al. 2014). Interestingly, the phosphorylation level of PIF4 increased under warm ambient temperatures, which stabilized the protein, in contrast to light-induced phosphorylation.  This suggests that light and temperature play an antagonistic role in regulating the activity of PIF4, at least moderately, through phosphorylation (Foreman et al. 2011). Warm temperature induces the translocation of BR-regulated transcription factor brassinazole-resistant 1 (BZR1) to the nucleus, where it binds to the promoter of PIF4 to promote expression and cell elongation (Ibañez et al. 2018). Recent studies have shown that the activity of MAP4K/target of temperature 3 (TOT3) kinase is required for thermomorphogenesis in both dicots and monocots. Compared to Col-0,  a lack of full-length TOT3 mutants resulted in a significantly shorter hypocotyl at 28°C. The TOT3 protein variants that lacked the kinase domain, which attenuates kinase activity, could not rescue defective phenotype at warm temperatures (Dai et al. 2021). Furthermore, TOT3 functions independently apart from PIF4 and phyB, but likely controls brassinosteroid-mediated hypocotyl growth by regulating BZR1 activity under warm temperatures. However, further study is required to determine how TOT3 controls BZR1 activity (Fig. 3).

In summary, phosphorylation is controlled by the balance between kinases and phosphatases in the regulation of protein activity. Under warm and extra high temperatures, there are different targets for kinases that respectively regulate multiple temperature signaling pathways for plant temperature response. However, the molecular mechanisms used by kinases and phosphatases that function under high temperature to (de)phosphorylate different targets are still unknown.

## Dynamic histone acetylation precisely controls gene expression  during the response to high temperatures

Chromatin structure has a dominant role in the regulation of gene expression and is modified through nucleosome positioning, histone variants, and post-translational modification of histones. Histone acetylation is a crucial modification in gene expression, and the histone acetylation level is mediated through histone acetyltransferases (HATs) and deacetylases (HDACs). The *Arabidopsis* genome contains 12 HAT genes, of which GCN5 is an intrinsic HAT that is involved in numerous plant development pathways and responses to stress. There were 16 kinds of HDACs have been identified in *Arabidopsis thaliana* (Shen et al. 2019). Increasing evidence shows that PTMs of histones are necessary for plants to adapt to environmental stress. Below, we summarize the roles of post-translationally modified histones in plant thermal responses.

**Fig. 4:**
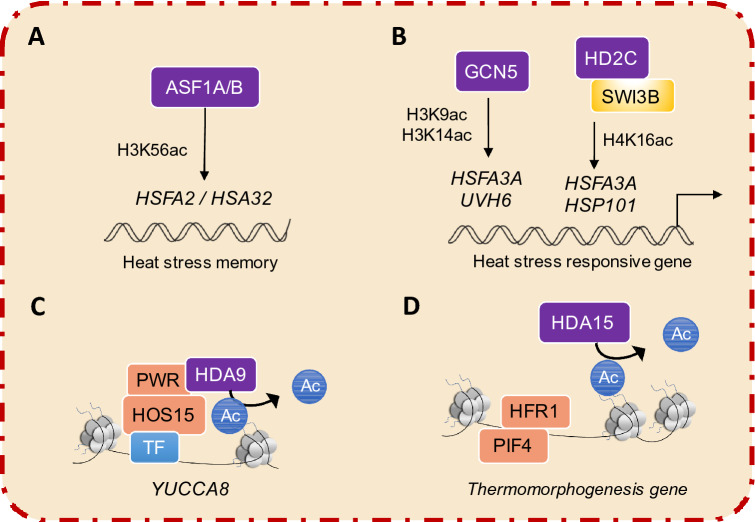
Histone modification in the plant thermal response. **A** ASF1A/B activates HSFA2 and HSA32 by facilitating H3K56ac. HSFA2 promotes transcriptional memory by positively influencing H3K4ac1/2/3. **B** Under HS, GCN5 regulates *HSFA3*, and *HSP101* transcription by facilitating H3K9ac and H3K14ac. Additionally, the histone deacetylases HD2C interacts with SWI3B to regulate heat stress response genes by altering H4K26ac levels. **C** Under warm temperatures, HOS15 interacts with histone deacetylase to form the HDA9-PWR-HOS15 complex that performs hypoacetylation at *YUCCA8* to regulate downstream genes. **D** HDA15 decreases the association with HFR1, which could compete with PIF4 to affect the expression of thermoresponsive genes that are repressed by HDA15

*Arabidopsis* ASF1 (ANTI-SILENCING FUNCTION 1) histone chaperones consist of ASF1A and ASF1B, which are associated with basal and acquired thermotolerance. With nucleosome removal and H3K56ac stimulation, they activate the transcription of the HSFA2 and HSA32 genes, implying that histone modification may be the key action in mediating the heat-stress response in plants (Weng et al. 2014) (Fig. 4A). Incapacitation of GCN5 represses the expression of heat-stress responsive genes, leading to further severe defects in terms of *Arabidopsis* thermotolerance. In particular, GCN5 is enriched in the promoter regions of *HSFA3* and *UVH6*, which regulate the H3K9 and H3K14 acetylation states. In addition, TaGCN5 in wheat can restore the defect in the thermotolerance phenotype in *Arabidopsis* *gcn5* mutant plants, suggesting that GCN5-mediated thermotolerance may be conserved in plants (Hu et al. 2015). Furthermore, HD2C, the well-studied HD2-type histone deacetylase (HDAC), directly interacts with the SWI/SNF complex subunit SWI3B, which is involved in the *Arabidopsis* response to heat stress by regulating the expression of heat-responsive genes and the H4K16Ac levels of *HSFA3* and *HSP101* (Buszewicz et al. 2016) (Fig. 4B).

Histone modification is also involved in plant thermomorphogenesis. The deacetylase HAD9 interacts with POWERDRESS (PWR), a SANT-domain containing protein, and promotes histone H3 deacetylation at specific genomic sites and is also involved in plant developmental processes, such as *Arabidopsis* aging (Chen et al. 2016a; Kim et al. 2016). Interestingly, PWR acts as a positive regulator for thermomorphogenesis and is necessary to deacetylate PIF4 and YUCCA8 (YUC8). In addition, histone H2A.Z dynamics on downstream genes are regulated by PWR, suggesting a potential link between histone deacetylation and H2A.Z nucleosome processes in regulating gene expression (Tasset et al. 2018). Further studies showed that the HDA9-mediated deacetylation of YUC8 by H3K9K14 permits PIF4 binding to the YUC8 promoter to induce YUC8 transcription, and is followed by auxin biosynthesis during thermomorphogenesis. Accordingly, the net eviction of H2A.Z from YUC8 nucleosomes is mediated through HDA9 activity at warm temperatures (Van Der Woude et al. 2019). Therefore, HDA9 and PWR are required for the heat-induced transcription of YUC8 and thermomorphogenesis. The HDA9 and PWR have partial redundancy of functions, but the function of PWR is more pleiotropic than that of HDA9 (Mayer et al. 2019). HOS15 is a core member of the HDA9-PWR complex, and mutated HOS15 alters global histone acetylation, which is similar to *hda9* and *pwr* mutants. However, the mechanism of HDA9-PWR-HOS15, a core histone deacetylase complex in plant thermo-morphogenesis, remains yet unknown (Mayer et al. 2019)(Fig. 4C). Deacetylase HDA15 interacts with the transcription factor HFR1 to regulate the gene expression involved in plant thermomorphogenesis, indicating that HDA15 may be recruited by the HFR1 to the chromatin to epigenetically repress downstream target genes during the plant response to elevated ambient temperature (Shen et al. 2019) (Fig. 4D). Therefore, HDA15 and HDA9 function in plant thermomorphogenesis by interacting with transcription factor HFR1 or PWR to regulate the acetylation level of downstream genes, indicating that HDA15 and HDA9 possess diverse functions in response to elevated temperature.

Overall, (de)acetylation is important for histone recruitment and their interaction with TFs for the modulation of stress-response gene expression under warm- or high- temperature stress.

## Histone methylation contributes to somatic and transgenerational thermomemory

The most abundant histone PTM is methylation, which usually occurs at the lysine and arginine residues of core histone tails. Mono-, di- and trimethylation in these residues at the N-terminal tails of H2A, H2B, H3, and H4 can further increase the indexing potential. In *Arabidopsis*, genome-wide analyses indicated that tri-methylation at H3K4 and H3K36 is generally recruited at actively transcribed genes, while other incidences of tri-methylation are associated with repressed genes or constitutive heterochromatin and silenced transposons. The precise role of histone arginine methylation has not been completely elucidated. However, because the levels of symmetric H3R2me2 and H4R3me2 negatively corresponded to the level of H3K4me3, an important marker for active transcription, it is possible that H3R2me2 and H4R3me2 could be involved in gene repression (Guccione et al. 2007; Zhao et al. 2009).

Accumulating evidence suggests that the modification of histones by methylation plays a vital role in plant thermomemory and thermotolerance. The levels of H3K4me3 and H3K4me2 at the specific loci of APX2 and HSP22.0 increased during acquired thermotolerance (heat stress memory). This depends on the HSF transcription factor HSFA2 that transiently associates with the promoters of these loci, suggesting that HSFA2 may recruit H3K4 histone methyltransferases to memory loci to modulate their sustained induction (Lämke et al. 2016). In addition, *HSFA2* is activated by heat-induced demethylation of H3K27me3, and in turn, positively regulates the expression of *REF6*. Therefore, HSFA2 and REF6 form a positive feedback loop to mediate transgenerational thermomemory in *Arabidopsis* (Liu et al. 2019). H3K36me3 also plays a key role in splicing regulation in response to fluctuating ambient temperature. It affects the splicing of upstream regulators of flowering time genes, such as *VIL2*  and *MAF5*, and thus, further affects temperature-induced flowering. However, the molecular mode of action still remains to be elucidated (Pajoro et al. 2017). Importantly, methylation of histones also plays a key role in crop response to heat stress. In rice, the levels of DNA and H3K9 methylation associated with OsFIE1 (Fertilization-Independent Endosperm 1) were decreased at 48 h and 72 h after fertilization under moderate heat stress, suggesting that epigenetic regulation partially contributes to the thermal sensitivity of endosperm development (Folsom et al. 2014). This result might provide new insights to increase crop thermotolerance.

To date, in plant studies, histone methylation changes via chromatin remodeling are required for development, stress tolerance, and short-/long-term memory. Bobadilla et al. presented a preliminary model for histone methylation changes in stress responses: before stress occurs, histone methylation limits the spreading of repressive chromatin marks and/or potentiates rapid transcriptional induction upon need at stress-related genes. Under stress, histone methylation is transiently induced from an inactive or a permissive chromatin state, to support the transcriptional initiation of stress-responding genes. Finally, histone methylation contributes to the accumulation of somatic memory or transgenerational memory (Bobadilla and Berr 2016).

## Conclusion and future directions

A change in temperature elicits signaling pathways in plants via perturbation of cellular homeostasis. Recently, there has been increased interest in shifting the researched focus from the single level of molecular regulation of stress-related gene expression to regulation in multiple dimensions, such as post-transcriptional and post-translational processes. In plants, alternative splicing (AS) regulation is a universal heat HSR mechanism. Some key HSR regulators, including HSF (Liu et al. 2013; Jang et al. 2014; Hu et al. 2020), bZIP (Cheng et al. 2015), DREB (Matsukura et al. 2010), and HSP (Ling et al. 2018), are precisely controlled in an HS-dependent manner by AS in response to heat stress and HS memory (Ling et al. 2018, 2021). Small RNAs (sRNAs) are also involved in heat stress tolerance in plants. MicroRNA398 is induced by HS and promotes the accumulation of HSF proteins, which enable heat stress tolerance in *Arabidopsis* (Chuck et al. 2007). MicroRNA396 targeted to HaWEKY6 regulates an early HS response in sunflower (Giacomelli et al. 2012). After the plant temperature increases or decreases, PTMs enable a more flexible mechanism for plants to activate networks of signaling events that promote resistance in short times in comparison to post-transcriptional processes. Thus, illustrating the mechanisms elaborately regulated by PTMs is crucial for plant adaptation to stressors and enhancement of crop resistance.

In plants, PTM of proteins is predominantly involved in mediating heat stress responses. There has been increased interest in this area of research. To date, although several reports have revealed the critical importance of PTM in plant heat-stress responses, the types of PTMs examined in high-throughput proteomics research are still very limited to several normally studied PTMs, such as the previously mentioned ubiquitination, SUMOylation, phosphorylation, acetylation, and methylation. These PTMs can induce protein conformational changes, or add and/or mask the interaction surfaces of targets resulting in sub-cellular re-localization, changes in enzymatic stability/activity, or interaction with other proteins, which expand the diversity and functionality of the proteome. To ensure that the signaling process is accurate, adding or removing PTMs to targets at specific sites requires specific enzymes, which are called "writers" and "erasers" of PTMs. The readers can recognize targets, writers, and erasers, which add or move the "signal" that changes the biological pathways. For this objective, studies were performed on genetic manipulation achieved by heterologous expression and natural/induced diversity. Negative mutations or overexpression of specific components in post-transcriptional or post-translational systems may generate genotypes with increased tolerance. For example, overexpression of RING finger E3 ligase OsHTAS, OsHCI1, or OsHIRP1 escalated heat tolerance in rice (Kim et al. 2020; Lim et al. 2013; Liu et al. 2016). Over-expression of the rice SUMO E3 ligase OsSIZ1 also enhanced plants subjected to heat stress and increased the photosynthesis rate (Mishra et al. 2018). Therefore, the developments in natural/induced diversity are very promising for crop improvements. During the last 5 years, CRISPR-associated (Cas) system-based genome editing for crops has been a fundamental breakthrough technique (Jaganathan et al. 2018). Although there has been little application of the technology to plants in the PTM field, CRISPR/Cas9 can be used for the generation of mutants with point mutations targeting amino acids in substrates to elucidate PTMs changes and their functions (Sander and Joung 2014). Combined with genome re-sequencing programs and mass spectrometry, it could allow the identification of the potential targets and creation of new alleles, which then could be used in the molecular breeding of new and improved plant genotypes in the future.

It is noteworthy that among high-temperature transcriptional regulation, we found PTM crosstalk, which switches the activation/attenuation of the same pathway, and ensures rapid regulation of signal transduction. PTM crosstalk is usually highly dynamic and relies on a refined spatial and temporal experimental backdrop. The crosstalk in phosphorylation–ubiquitination–SUMOylation has been identified (Dai et al. 2018). For example, the regulation of DREB2A and PIF4 is involved in the heat stress response under HS by three types PTMs crosstalk (Mizoi et al. 2019; Morimoto et al. 2017; Wang et al. 2020). Ubiquitination–SUMOylation crosstalk has two sides. SUMOylation nearly sustains the stability of targets occupied by lysine for inhibited protein degradation. In the typical example from COP1 and SIZ1, SUMOylation or ubiquitination dynamically regulates high-temperature-induced growth responses. However, SUMO can undergo regulated proteolysis initiated by the formation of a poly-SUMO chain through the activity of SUMO ligases (E4), such as PIAL1/2. This chain recruits STUbLs, which attach poly-ubiquitin chains (Ub) to SUMO and the target protein so that they can then be degraded by the 26S proteasome. This introduces another layer of transcriptional regulation (Wawrzyńska and Sirko 2020). However, the overall orchestration and possible crosstalk of these PTMs in plant stress-response regulation remain unexplored.

The investigation of PTM response to temperature in plants has thus far mainly focused on a single target and enzyme. The molecular mechanism remains to be understood. First, how to recognize a heat signal remains a crucial topic that requires exploration. Previous research has revealed that AtCaM interacts with protein kinase to regulate the phosphorylation status of HSFA1. It is unclear whether calcium signals function as primary heat sensors. Second, it is unknown how the post-translational related factor is recruited to target a gene locus to modulate gene expression. These issues require elucidation in the future, and further data will provide novel insight into improving thermotolerance in crops.
